# Syringe-Assisted Arthroscopic Implantation of Particulated Juvenile Articular Cartilage for Trochlear Cartilage Defects

**DOI:** 10.7759/cureus.110235

**Published:** 2026-06-04

**Authors:** Andy Contreras, Dhruv Limaye, Christina Im, Olore Imoohi, Timothy P Liu, Al-Hassan J Dajani, Thomas J Kremen

**Affiliations:** 1 Orthopedic Surgery, UCLA Medical Center, Los Angeles, USA

**Keywords:** arthroscopy, cartilage restoration, dry arthroscopy, fibrin sealant, focal chondral defect, particulated juvenile articular cartilage, patellofemoral joint, trochlea, trochlear cartilage defect, tuberculin syringe

## Abstract

Particulated juvenile articular cartilage (PJAC) allograft has emerged as a single-stage biologic option for treatment of focal full-thickness cartilage defects, including isolated trochlear lesions. Open techniques are most commonly described, and although a small number of arthroscopic techniques have been reported, precise placement of particulate graft material within the curved geometry of the trochlea remains technically challenging due to high local shear forces, limited working angles, and difficulty maintaining a dry arthroscopic field. We describe a fully arthroscopic technique for the implantation of PJAC allograft in a trochlear cartilage defect using a standard 1-mL disposable tuberculin syringe to facilitate controlled, titratable graft delivery and uniform particulate distribution. The technique is demonstrated in a representative case and includes defect preparation with creation of stable vertical cartilage shoulders, removal of the calcified cartilage layer, transition to a dry arthroscopic field, syringe-assisted graft placement, and stabilization with fibrin sealant. Syringe-assisted delivery enabled controlled placement of cartilage particulates with uniform distribution under direct arthroscopic visualization, facilitating creation of a thin monolayer flush with the surrounding articular surface. The patient initially progressed through rehabilitation without wound or infectious complications and maintained full knee motion. Postoperative magnetic resonance imaging demonstrated incorporation of the lateral trochlear PJAC implant without delamination or subchondral bone plate disruption. In the setting of a complex prior surgical history, multifocal patellofemoral chondral disease, and persistent activity-related mechanical symptoms, the patient later underwent repeat diagnostic arthroscopy approximately 16 months after PJAC implantation. A standard disposable tuberculin syringe provides a simple, reproducible, and cost-efficient method for improving graft control during arthroscopic PJAC implantation in the trochlea, leveraging widely available instrumentation rather than specialized delivery systems. This technique may help reduce technical barriers to minimally invasive cartilage restoration in anatomically constrained regions of the patellofemoral joint.

## Introduction

Particulated juvenile articular cartilage (PJAC) allograft transplantation has emerged as a single-stage biologic option for the treatment of focal full-thickness cartilage defects [1]. PJAC consists of minced fragments of juvenile donor cartilage containing viable chondrocytes embedded within native extracellular matrix, allowing implantation without cell culture or a staged harvest [1]. The rationale for juvenile cartilage is supported by basic science data demonstrating enhanced regenerative potential compared with adult cartilage, including increased cellularity, proliferative capacity, and extracellular matrix production that may promote hyaline-like repair tissue formation [2-4]. Clinical investigations have further demonstrated favorable outcomes following PJAC implantation for patellofemoral cartilage defects in young patients, including improved return-to-sport rates, patient-reported outcome scores, and defect fill on postoperative magnetic resonance imaging (MRI) [5].

Although focal cartilage restoration techniques continue to evolve, managing trochlear defects remains technically challenging because of high local shear forces, the complex geometry of the patellofemoral joint, limited arthroscopic working angles, and the importance of creating a flush articular surface [6]. Prior literature has described open approaches for PJAC implantation in the knee, as well as arthroscopic PJAC techniques in other anatomically constrained joints such as the talus [5,7,8]. Additionally, PJAC has been combined with bone grafting in osteochondral lesions requiring restoration of subchondral bone support [9]. Arthroscopic PJAC implantation in the trochlea has also been reported, although these previously described techniques rely on alternative delivery methods that may limit precise graft placement [1]. Collectively, these reports support the feasibility of arthroscopic PJAC as a single-stage cartilage restoration strategy in the trochlea, while highlighting that controlled delivery and uniform distribution of particulate graft material remain key technical challenges.

Arthroscopic implantation of PJAC is a promising approach that offers advantages including minimized soft-tissue disruption, preservation of surrounding structures, and enhanced visualization of defect preparation and graft contouring [7]. However, previously described arthroscopic PJAC techniques have also emphasized important technical challenges, particularly in anatomically constrained regions such as the trochlea [7,9]. These challenges include limited working angles within the patellofemoral compartment, difficulty maintaining a consistently dry arthroscopic field, and the technical burden associated with handling and evenly distributing individual particulate graft fragments using standard arthroscopic instruments [7]. In these settings, repeated manipulation of individual graft fragments can increase the difficulty of achieving uniform particulate distribution, and the curved geometry of the trochlear surface may increase the risk of graft dispersion or migration before fibrin stabilization [9]. A delivery method that allows gradual, titratable extrusion of PJAC directly into the prepared defect may therefore improve graft control while reducing the need for fragment-by-fragment arthroscopic handling.

This technical report describes a fully arthroscopic technique for PJAC implantation in a trochlear cartilage defect using a standard disposable tuberculin syringe for graft delivery. This approach builds upon previously described arthroscopic PJAC techniques [7,9] by providing a simple, low-cost, and readily available method that facilitates more controlled, titratable extrusion and uniform distribution of particulate graft material under direct visualization. By addressing limitations related to graft handling and particulate dispersion in confined working spaces, this technique may improve reproducibility and graft control during arthroscopic cartilage restoration in anatomically constrained regions of the knee.

## Technical report

Patient selection

Arthroscopic PJAC implantation is considered for symptomatic, focal full-thickness cartilage defects (International Cartilage Repair Society grade 3 or 4) [10] with minimal subchondral bone loss and adequate surrounding cartilage shoulders. Patients should have acceptable alignment and stability, with meniscal integrity sufficient to protect the involved compartment. Advanced degenerative changes, uncorrected malalignment or instability, and substantial subchondral bone loss are addressed with alternative or concomitant procedures.

Preoperative imaging and planning

**Figure 1:**
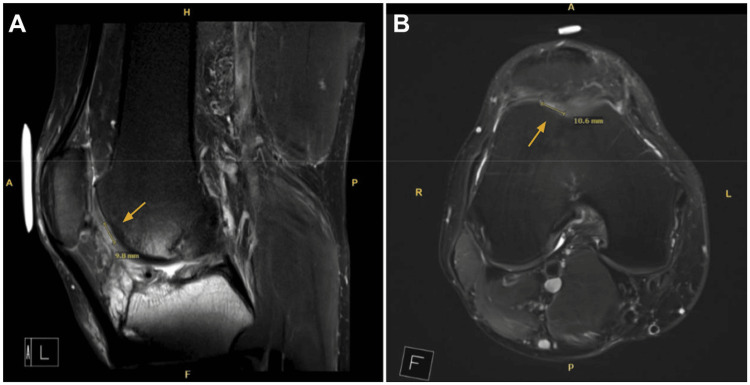
Preoperative magnetic resonance imaging of the right knee in a patient with a focal full-thickness chondral defect of the lateral trochlea. (A) Sagittal image with orange arrow indicating the defect, measuring approximately 1 cm in the anteroposterior dimension. (B) Axial image with orange arrow indicating the defect, measuring approximately 1.1 cm in the transverse dimension. Yellow labels denote anatomic orientation (A, anterior; H, head; F, foot; P, posterior; R, right; L, left).

Patients should undergo a comprehensive physical examination, followed by imaging studies. Standard radiographs, including alignment films, should be obtained to evaluate for fracture, advanced osteoarthritis, or malalignment. MRI is essential to identify concomitant intra-articular pathologies that may require concurrent surgical management or represent contraindications to the procedure. Additionally, MRI provides a detailed characterization of the osteochondral lesion, aiding in surgical planning. The MRI of the lesion treated in this technique article is presented in Figures 1A, 1B.

Patient positioning and portal placement

The procedure is performed under general anesthesia with the patient positioned supine on the operating table. After induction of anesthesia, an examination under anesthesia is performed to assess knee stability and range of motion. The operative extremity is prepped and draped in standard sterile fashion. Prophylactic intravenous antibiotics are administered prior to incision.

Defect preparation

**Figure 2:**
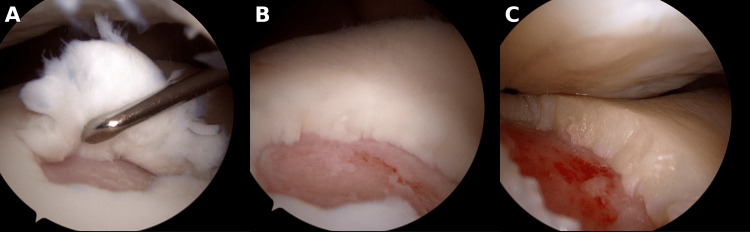
Arthroscopic progression of the trochlear chondral lesion. (A) Arthroscopic view of the trochlear cartilage lesion with an unstable flap prior to debridement. (B) Trochlear articular cartilage lesion following flap debridement. (C) Prepared defect following debridement, demonstrating stable margins and removal of the calcified cartilage layer.

Standard anterolateral and anteromedial parapatellar portals are established, and diagnostic arthroscopy is performed using a 30-degree arthroscope to systematically evaluate all compartments of the knee. In this representative case, prior cartilage restoration within the patellofemoral joint is inspected and found to be well incorporated, without evidence of delamination, overgrowth, or instability. A focal, full-thickness chondral defect with an associated unstable cartilage flap is identified on the lateral trochlea (Figure 2A). The surrounding cartilage margins are stable, and the cruciate ligaments are intact.

Graft preparation

Attention is then directed to preparation of the trochlear defect. Unstable cartilage was excised using a combination of an arthroscopic biter, grasper, and ring curette until stable, well-defined vertical margins are achieved (Figure 2B). The calcified cartilage layer is meticulously removed with a ring curette to expose a healthy subchondral surface while avoiding violation of the underlying bone. The defect is probed to confirm complete removal of unstable tissue and adequacy of preparation (Figure 2C).

Dry arthroscopy and graft delivery

**Figure 3:**
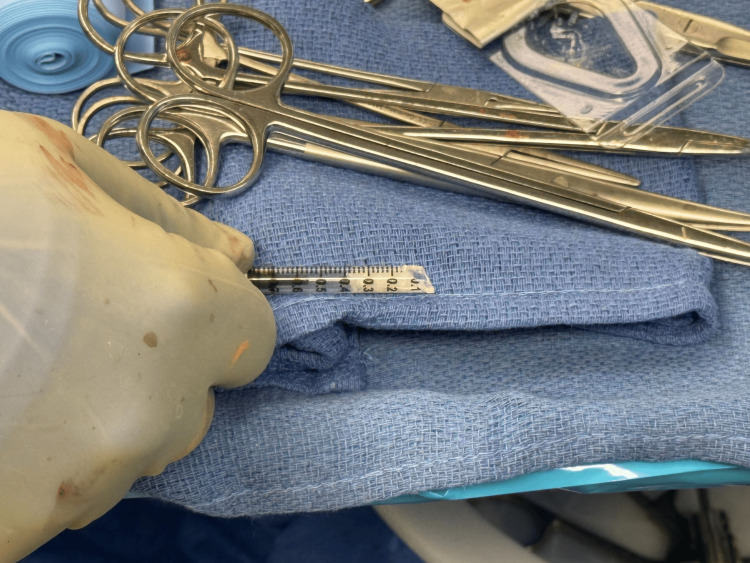
Back-table preparation of the disposable 1-mL tuberculin syringe used for arthroscopic PJAC delivery. The distal tip of the syringe has been trimmed with a sterile scalpel to widen the aperture and facilitate controlled extrusion of the cartilage particulates. PJAC, particulated juvenile articular cartilage

The PJAC allograft (DeNovo NT; Zimmer Biomet, Warsaw, IN, USA) is prepared on the back table. The graft is removed from its packaging, excess fluid is evacuated, and the cartilage particulates are loaded into a standard 1-mL disposable tuberculin syringe (Figure 3).

**Figure 4:**
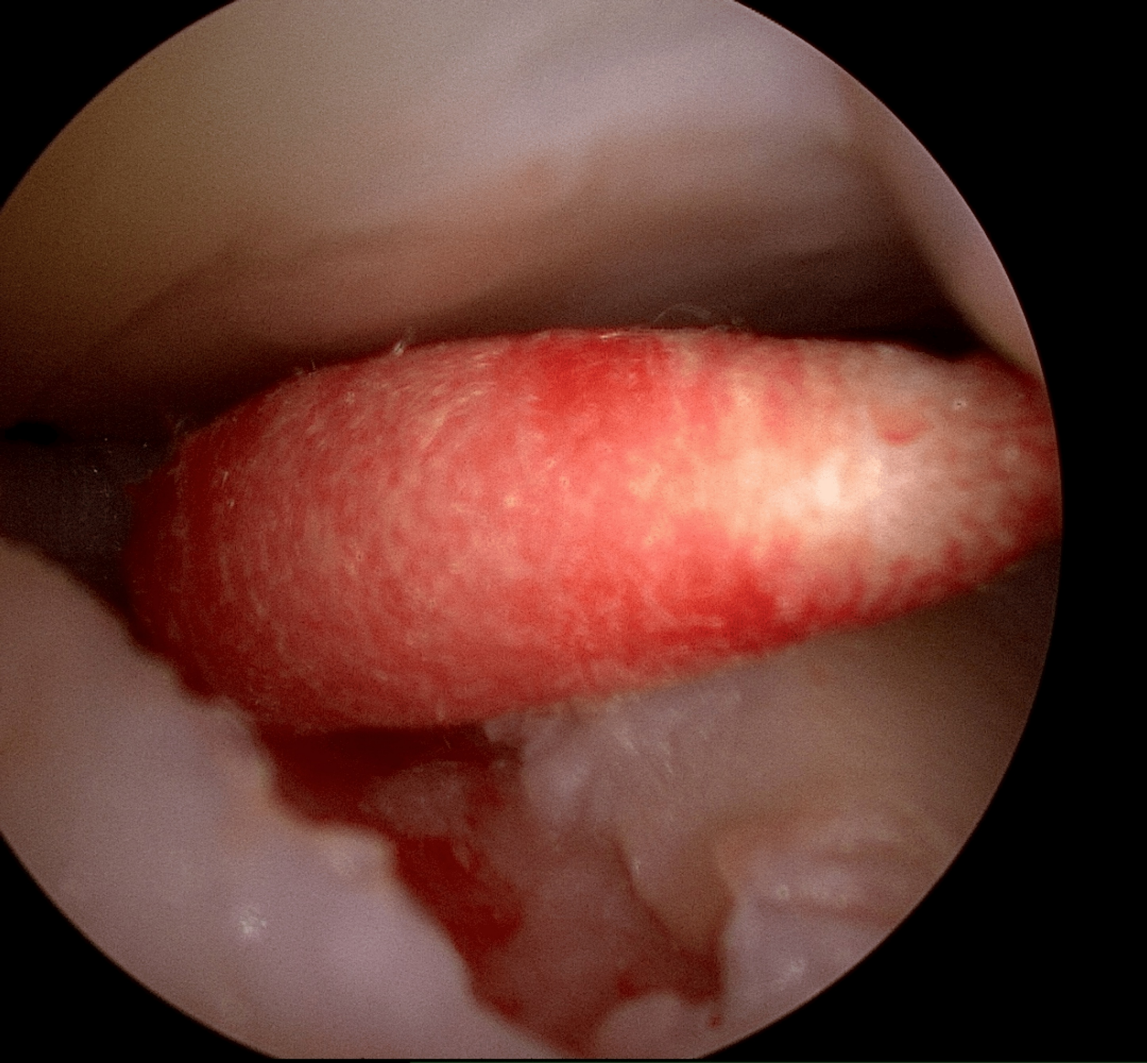
Arthroscopic visualization of the trochlear chondral defect during dry arthroscopy. A sterile cotton-tipped applicator is utilized to remove residual fluid and achieve a dry recipient bed, facilitating improved graft adherence during subsequent cartilage implantation.

Fibrin glue (Tisseel®; Baxter Healthcare, Deerfield, IL, USA) is prepared according to the manufacturer's instructions. Arthroscopic fluid is then evacuated from the joint to transition to a dry arthroscopic environment. The defect bed is gently dried using a sterile cotton swab under direct visualization (Figure 4).

**Figure 5:**
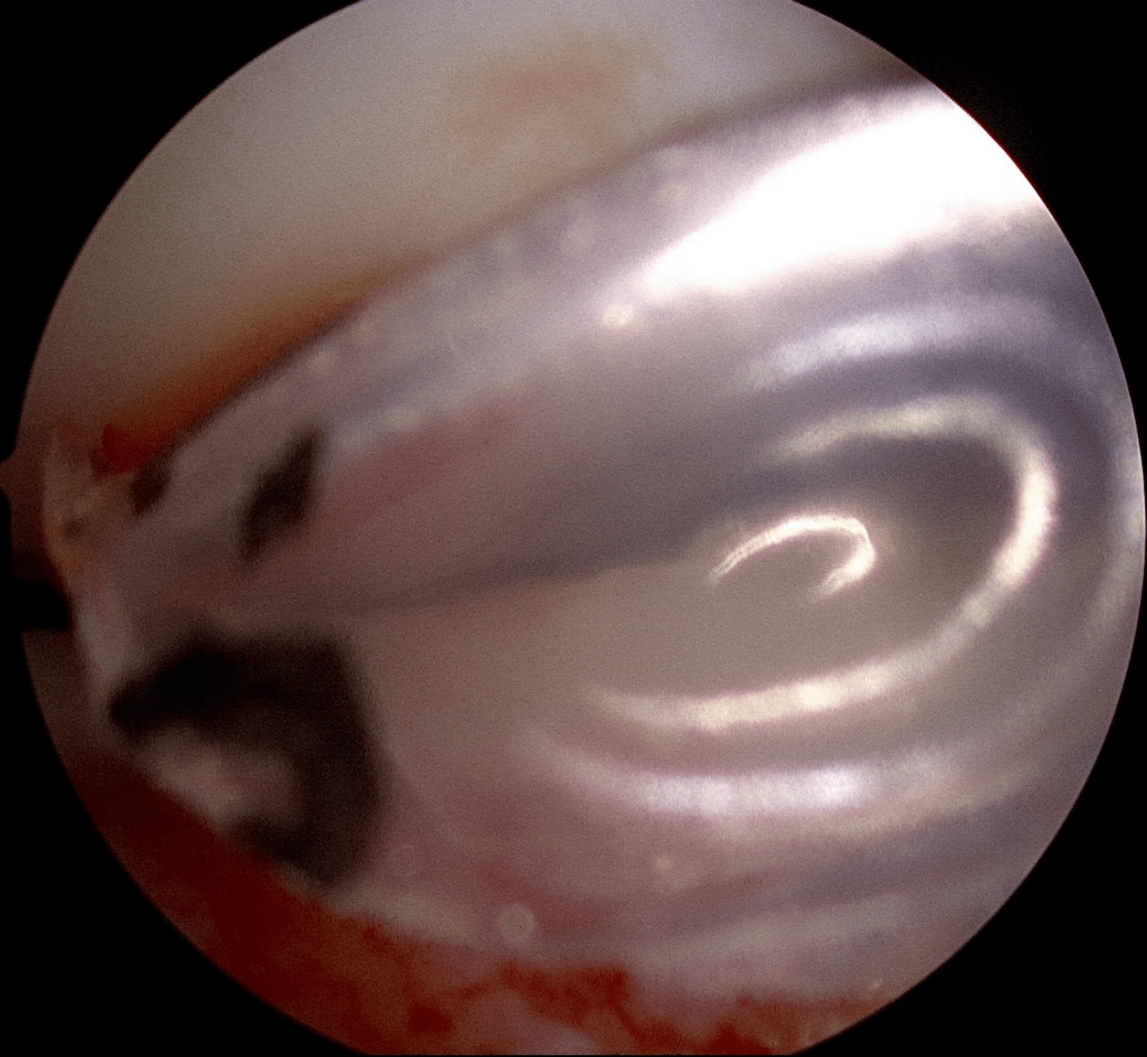
Arthroscopic view of the 1-mL tuberculin syringe delivering the PJAC. The syringe allows controlled placement of the material directly into the prepared trochlear defect, facilitating precise placement and contouring within the lesion. PJAC, particulated juvenile articular cartilage

A thin base layer of fibrin glue is applied arthroscopically to the prepared trochlear defect using the manufacturer's dual-syringe delivery system. The PJAC-loaded tuberculin syringe is introduced through the medial portal, and the cartilage particulates are slowly expressed into the defect under direct arthroscopic visualization (Figure 5).

**Figure 6:**
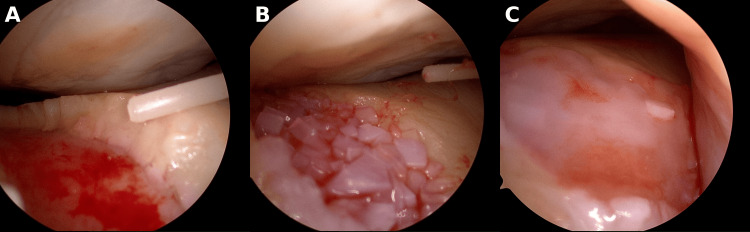
Arthroscopic implantation of PJAC in the trochlea. (A) Prepared full-thickness trochlear chondral defect following debridement of the calcified cartilage layer, ready for implantation. (B) Trochlear defect following the placement of PJAC via a 1-mL disposable tuberculin syringe within the defect under direct arthroscopic visualization. (C) Final construct after graft contouring and fibrin sealant stabilization, demonstrating a flush and stable repair. PJAC, particulated juvenile articular cartilage

Using a Freer elevator, the graft fragments are evenly distributed and gently compacted to create a uniform monolayer that conforms to the native trochlear contour and is flush with the surrounding articular surface (Figures 6A-6C).

Fibrin sealant stabilization

Once satisfactory graft fill and contour were achieved, a second thin layer of fibrin glue is applied over the graft to stabilize the construct. The fibrin glue is allowed to polymerize for approximately three minutes without irrigation. After curing, gentle probing and passive knee range of motion are performed to confirm graft stability. All arthroscopic instruments are removed, and the portals are closed in standard fashion. Sterile dressings are applied, and a hinged knee brace locked in extension is placed on the operative extremity. Key technical pearls and common failure points are summarized in Table 1.

**Table 1:** Pearls and pitfalls of syringe-assisted arthroscopic PJAC trochlear implantation. This table was created by the authors and is original. PJAC, particulated juvenile articular cartilage

Step	Pearls	Pitfalls
Lesion assessment	Confirm the lesion is focal, contained, and accessible arthroscopically.	Proceeding with uncontained lesions increases risk of graft migration.
Defect preparation	Create stable vertical margins and remove the calcified cartilage layer.	Incomplete preparation compromises graft adherence.
Dry arthroscopy	Thoroughly evacuate fluid prior to graft placement.	Residual fluid prevents fibrin glue polymerization.
Fibrin base layer	Apply a thin, uniform layer.	Excess glue creates an uneven graft bed.
PJAC delivery	Use slow, controlled extrusion to form a uniform monolayer.	Overfilling results in a proud graft surface.
Graft contouring	Gently level particulates flush with surrounding cartilage.	Excess manipulation disrupts graft stability.
Fibrin cap	Apply a sealing layer and allow adequate set time.	Early knee motion risks graft displacement.

Postoperative course and early outcome

At the initial postoperative visit, the patient was healing appropriately without fever, wound drainage, or clinical evidence of infection. He was ambulating with crutches and a hinged knee brace and reported no catching, locking, swelling, or instability. He was advanced through a supervised rehabilitation protocol with toe-touch weight-bearing initially, progression of weight-bearing under physical therapy guidance, and discontinuation of the brace and crutches once active knee extension, quadriceps control, and gait control were restored.

At approximately five months postoperatively, the patient had full knee range of motion but reported persistent painful clicking during knee flexion and extension, swelling after longer-distance ambulation, and difficulty in deep squatting despite extensive physical therapy. MRI obtained approximately five months after surgery demonstrated incorporation of the lateral trochlear PJAC implant without delamination or subchondral bone plate disruption. The graft appeared attenuated compared with adjacent native cartilage and demonstrated incomplete fill with surface fissuring; however, no new high-grade patellofemoral cartilage defect was identified.

The patient’s postoperative course was interpreted in the context of a complex surgical history, including multiple prior ipsilateral knee procedures and prior patellofemoral stabilization and cartilage restoration procedures. At a later follow-up, he maintained knee range of motion from -5° to 140° without effusion, joint-line tenderness, ligamentous instability, or patellar apprehension, but continued to report intermittent pain and snapping with activity despite physical therapy and ultrasound-guided corticosteroid injection for suspected plica-related symptoms. Formal patient-reported outcome measures were not available for this representative technical case.

Given persistent mechanical symptoms and concern for synovitis, scar tissue, loose chondral tissue, or recurrent chondral pathology in a multiply operated patellofemoral joint, the patient underwent repeat diagnostic arthroscopy approximately 16 months after PJAC implantation. Arthroscopy demonstrated abundant synovitis, fibrotic bands, a loose body, multifocal patellofemoral chondromalacia, and a grade 3-4 chondral lesion at the lateral trochlea in the region of prior PJAC implantation with an unstable full-thickness flap extending centrally. Chondroplasty, synovectomy, and loose body removal were performed.

## Discussion

Rationale and novelty

There is limited literature describing arthroscopic PJAC implantation specifically in the trochlea. Existing reports primarily describe PJAC use for patellar lesions, as well as arthroscopic implantation techniques in other anatomically constrained regions such as the talus and tibial plateau [5,8]. Farr and Yao originally introduced PJAC as a single-stage option for focal cartilage repair and established foundational principles regarding defect preparation and graft stabilization [8]. Subsequent basic science investigations have supported the biologic rationale for juvenile cartilage, demonstrating increased cellularity, proliferative capacity, and extracellular matrix production compared with adult cartilage, which may contribute to hyaline-like repair tissue formation [2-4].

Comparison with prior techniques

The technique described in this article addresses a practical limitation of arthroscopic PJAC implantation in the trochlea: achieving controlled delivery and uniform distribution of particulate graft material within a curved patellofemoral surface. Use of a standard disposable tuberculin syringe allows gradual, titratable extrusion of PJAC fragments under direct visualization, facilitating precise placement and minimizing inadvertent graft dispersion prior to fibrin stabilization. This method builds upon previously described arthroscopic PJAC techniques while leveraging cost-efficient and readily available instrumentation to avoid specialized delivery systems [7,9].

Arthroscopic versus mini-open approach

A fully arthroscopic approach offers several potential advantages over mini-open cartilage restoration techniques. By avoiding a mini-arthrotomy, arthroscopy may reduce approach-related soft-tissue disruption while preserving the extensor mechanism and peripatellar tissues, which is particularly relevant for patellofemoral procedures. In comparative cartilage repair literature, arthroscopic approaches have demonstrated clinical outcomes comparable to mini-open techniques, supporting arthroscopy as a viable alternative when lesions are well-contained and accessible [7]. In addition, arthroscopy provides magnified visualization that facilitates precise defect preparation and controlled graft delivery, which may enhance procedural reproducibility while maintaining a minimally invasive surgical profile [11]. Although limited arthrotomy may still be required in select patellofemoral cases to optimize visualization or instrument trajectory, careful patient and lesion selection allows many trochlear defects to be addressed using an all-arthroscopic strategy [1,11].

Technical pearls and pitfalls

Several technical considerations are critical for successful graft implantation. Defect preparation should include creation of stable vertical cartilage shoulders and meticulous removal of the calcified cartilage layer to optimize graft adherence [1]. Transitioning to a dry arthroscopy environment is essential, as fluid ingress can disrupt particulate positioning and compromise fibrin fixation. The graft should be distributed in a thin, even monolayer that conforms to the native trochlear contour, followed by fibrin glue application and adequate polymerization time prior to range-of-motion testing. Key technical pearls and potential pitfalls associated with this technique are summarized in Table 1.

Limitations

This technical report has limitations inherent to its design, including description of a single representative case, the absence of formal patient-reported outcome measures, and limited ability to draw conclusions regarding clinical efficacy, as the primary aim is to describe technical feasibility and reproducibility of graft delivery. The postoperative course should also be interpreted in the context of the patient’s complex prior surgical history, multifocal patellofemoral chondral disease, and high-demand activity goals, which limit attribution of later symptoms to any single lesion or intervention. While prior clinical series have reported favorable short- and mid-term outcomes following PJAC implantation for patellofemoral and knee cartilage defects, including improvements in patient-reported outcomes and return-to-sport rates [5,11-13], longer-term durability, graft maturation characteristics, and comparative effectiveness remain areas of ongoing investigation [14-16]. Further studies are necessary to evaluate durability, graft maturation, and potential failure mechanisms following trochlear PJAC implantation. Future investigations should also clarify optimal indications for isolated chondral lesions versus osteochondral defects requiring concurrent bone grafting, as described in other anatomic locations.

## Conclusions

The syringe-assisted arthroscopic technique presented here offers a simple, reproducible, and cost-efficient approach for controlled PJAC delivery in select trochlear cartilage defects. By allowing titratable extrusion and uniform particulate distribution under direct arthroscopic visualization, this method may improve graft handling in anatomically constrained regions of the patellofemoral joint while avoiding specialized delivery instrumentation. Further clinical investigation with larger cohorts, standardized outcome measures, and longer follow-up is warranted to evaluate durability, graft maturation, and comparative outcomes.
